# Automated Analysis Using a Bayesian Functional Mixed-Effects Model With Gaussian Process Responses for Wavelet Spectra of Spatiotemporal Colonic Manometry Signals

**DOI:** 10.3389/fphys.2020.605066

**Published:** 2021-02-11

**Authors:** Lukasz Wiklendt, Marcello Costa, Mark S. Scott, Simon J. H. Brookes, Phil G. Dinning

**Affiliations:** ^1^College of Medicine and Public Health, Centre for Neuroscience, Flinders University, Bedford Park, SA, Australia; ^2^Centre for Neuroscience, Surgery and Trauma, Blizard Institute, Queen Mary University of London, London, United Kingdom; ^3^Discipline of Surgery and Gastroenterology, Flinders Medical Centre, Bedford Park, SA, Australia

**Keywords:** continuous wavelet transform, Bayesian mixed effects, Gaussian process, colonic manometry, spatiotemporal analysis

## Abstract

Manual analysis of human high-resolution colonic manometry data is time consuming, non-standardized and subject to laboratory bias. In this article we present a technique for spectral analysis and statistical inference of quasiperiodic spatiotemporal signals recorded during colonic manometry procedures. Spectral analysis is achieved by computing the continuous wavelet transform and cross-wavelet transform of these signals. Statistical inference is achieved by modeling the resulting time-averaged amplitudes in the frequency and frequency-phase domains as Gaussian processes over a regular grid, under the influence of categorical and numerical predictors specified by the experimental design as a functional mixed-effects model. Parameters of the model are inferred with Hamiltonian Monte Carlo. Using this method, we re-analyzed our previously published colonic manometry data, comparing healthy controls and patients with slow transit constipation. The output from our automated method, supports and adds to our previous manual analysis. To obtain these results took less than two days. In comparison the manual analysis took 5 weeks. The proposed mixed-effects model approach described here can also be used to gain an appreciation of cyclical activity in individual subjects during control periods and in response to any form of intervention.

## Introduction

Colonic manometry is a procedure involving the placement of a flexible catheter incorporating pressure sensors into the colon to record contractile activity. It has been used to distinguish normal colonic contractions in healthy adult subjects (Bassotti et al., 1987; Bassotti and Gaburri, 1988; Soffer et al., 1989; Bampton et al., 2001; Rao et al., 2001b) from the abnormal contractility that may exist in patients with functional colonic disorders (Narducci et al., 1986; Bassotti et al., 1988; Chey et al., 2001; Dinning et al., 2010). More recently, several research groups have published findings from high-resolution colonic manometry. These catheters utilize a greater number of more closely spaced recording sensors, that provide a clearer picture of propagating contractile activity (Dinning, 2018; Pervez et al., 2020).

**FIGURE 1:**
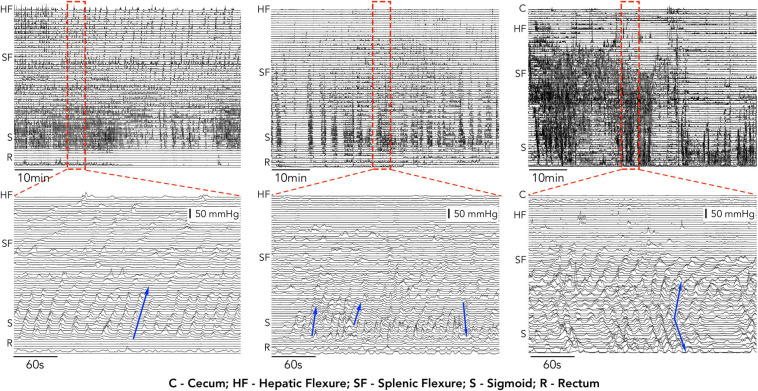
1 h, examples of high-resolution colonic manometry traces recorded in 3 of our subjects. Each example contains a high number of propagating motor patterns. Expanded regions from within the red hatched rectangle are displayed below each 1 h example. In these expanded regions individual propagating contractions can be identified (examples shown by blue arrows). The manual identification of each event, its start and end location and direction become time consuming and, in some cases, (middle and right) increasingly difficult.

Despite the improvements in catheter design, analysis of manometric recordings still relies upon either visual identification of propagating motor patterns or a generalized approach using area under the pressure curve (AUC) or motility index (MI) measurement. Visual identification of colonic motor patterns has identified differences in the count, velocity and amplitude of propagating pressure waves between health and patient groups, however, this approach is also subject to some fundamental problems. In some manometry traces the large number of pressure events can make identifying individual motor patterns very difficult. This is highlighted in Figure 1 which shows manometry traces recorded in 3 of our subjects. Not only is it time consuming to find each individual propagating event, but determining where they start, end and their direction of propagation can also be difficult.

Composite measures such as AUC or MI avoid visual identification of motor patterns, however, their non-specific nature makes useful interpretation of the data very limited. For example, an increase or decrease in AUC or MI, within or between subjects, tells us little about the altered characteristics of specific motor patterns.

Automated approaches that identify and quantify changes in motor patterns, standardize analysis between laboratories and remove potential personal bias, all within a workable time frame would be very beneficial to the international community. There have been attempts to achieve this previously (De Schryver et al., 2002; Pan et al., 2010; Wiklendt et al., 2013) but those developed techniques have not been adapted by any other groups. Part of the problem is the ability to determine the clinical worth of the findings from these automated approached. For example, in two approaches, the findings suggest disjointed or poorly coordinated pressure waves in patients with slow transit constipation when compared to healthy adults (Pan et al., 2010; Wiklendt et al., 2013). While of potential interest the analysis does not allow us to determine which pressure wave are poorly coordinated.

In this current article, we developed a computerized approach for the analysis of high-resolution colonic data. The technique is based upon a wavelet transform method, currently used in analyzing time-series in fields such as neuroscience, geophysics, meteorology and oceanography (Torrence and Compo, 1998; Grinsted et al., 2004; Veleda et al., 2012). The wavelet transform is a signal processing technique that can be used to transform signals from the time domain to the time-frequency domain, effectively decomposing them into constituent frequencies. Using this approach, we are able to see in a single image changes in colonic pressure waves, at all frequencies, in response to any given stimulus (a meal in this instance). The images also contain information on propagation direction and speed of propagation and the statistical comparisons to determine if any stimulus effects differ between subject groups. We have applied this analytical method to data that we had previously analyzed manually in healthy adults (Dinning et al., 2014) and patients diagnosed with slow transit constipation (Dinning et al., 2015). The findings in that original article are compared to the findings from our developed automated approach in the section “Discussion.”

The structure of the article is as follows. Section “Spectral Decomposition” describes spectral decomposition with the wavelet and cross-wavelet transforms. Section “Statistical Framework” details the statistical framework that is used to compare the spectra between groups of subjects. We present an application of this technique to colonic manometry data described in Section “Data,” with results shown in Section “Results.” The article concludes with a discussion in Section “Discussion.”

## Spectral Decomposition

### Wavelet Transform

The continuous wavelet transform (Torrence and Compo, 1998; Mallat, 2008) is a useful tool for analyzing non-stationary quasiperiodic signals. It decomposes a time domain signal *x*(*t*) ∈ *ℝ* into the time-scale domain *w*(*t*,*s*) ∈ *ℂ* with equation (2.1):

(2.1) $$w\,\left(t,s\right)=\int_{-\infty }^{\infty }x\,\left(\tau \right)\,\frac{1}{\sqrt{s}}\,\psi^{*}\,\left(\frac{\tau -t}{s}\right)\,d\tau$$

where ψ(*t*) ∈ *ℂ* is an admissible *wavelet* function, and the * superscript represents the complex conjugate. An admissible wavelet function is one which has zero mean and its Fourier transform is continuously differentiable (Farge, 1992), with an extra desirable property that it be localized in both time and frequency. Intuitively, *w*(*t*,*s*) measures the variation of *x*(*t*) within a neighborhood at *t* of size proportional to *s*.

In practice, we choose *s* from finite set of logarithmically spaced scales *S* = {*s*_1_,…,*s*_*L*_}, specify the wavelet basis function in the frequency domain, and perform the convolution in equation (2.1) via fast Fourier transform (FFT) utilizing the convolution theorem with:

(2.2) $$w\,\left(t,s\right)={ℱ}^{-1}\,\left[X\,\left(\omega \right)\,\sqrt{s}\,\Psi^{*}\,\left(s\,\omega \right)\right]\,\left(t\right)$$

where *s* ∈ *S*, Ψ = *ℱ*[ψ] is frequency-domain wavelet function, *X* = *ℱ*[*x*] is the frequency-domain signal, *ℱ* and *ℱ*^−1^ are the Fourier and inverse Fourier transforms, and ω represents the frequency-domain locations in radians per second. The wavelet transform is susceptible to harmonic artifacts, and we solve this problem by applying the “MesaClip” algorithm as described in our recent article (Wiklendt et al., 2020).

To map from scales (seconds) to frequencies (Hz) we use “Synchrosqueezing” (Daubechies et al., 2011). Synchrosqueezing redistributes the wavelet coefficients based on the first time-derivative of the phase (also known as “instantaneous frequency”). For a given set of *K* equally and logarithmically spaced frequency bins with centers *F* = {*f*_1_,…,*f*_*K*_}, synchrosqueezing can be described as:

(2.3) $$v\,\left(t,f\right)=\sum_{s\in S}\frac{w\,\left(t,s\right)}{\sqrt{s}}\,{\text{bin}}_{f}\,\left(\frac{1}{2\,\pi }\,\frac{\partial \phi \,\left(t,s\right)}{\partial t}\right)$$

where *ϕ*(*t*,*s*) = unwrap(∠*w*(*t*,*s*)) represents the time-differentiable “unwrapped-in-time” phase in radians with the complex argument (or angle) denoted by the parentheses-less function ∠:*ℂ*→(−π,π]. The function bin_*f*_(*x*) returns *1* if *x* and *f* are in the same bin, and *0* otherwise.

Switching to discrete-time representation with samples recorded at times *T* = {*t*_1_,…,*t*_*N*_} we can view the wavelet spectrum as *v*(*t*,*f*):*T*×*F*→*ℂ*. The time-average of the squared amplitudes produces the global wavelet power spectrum:

(2.4) $$\hat{v}\,{\left(f\right)}^{2}=\frac{1}{N}\,\sum_{t\in T}{\left|v\,\left(t,f\right)\right|}^{2}$$

### Cross-Wavelet Transform

The cross-wavelet transform combines two wavelet spectra with the complex-conjugated product:

(2.5) $$v_{a\,b}\,\left(t,f\right)=v_{a}\,\left(t,f\right)\,v_{b}^{*}\,\left(t,f\right)$$

where *v_a* and *v_b* are the synchrosqueezed wavelet transforms of the two signals labeled *a* and *b*. The combined subscript *v*_*ab*_ denotes the cross-wavelet transform between the two signals.

A global wavelet power cross-spectrum could be computed in the same way for *v*_*ab*_ as shown for *v* in equation (2.4). However, this discards the useful phase information contained in *v*_*ab*_. The effect of the complex-conjugated product is that the resulting phase represents the difference in phase between the two signals. For each frequency, computing a squared-amplitude-weighted histogram of the phase-differences yields a 2D histogram in the frequency-phase domain, analogous to the global wavelet power spectrum but stratified by phase-differences.

Since phase-differences are actually phases, in the rest of this section we will refer to them simply as “phases,” keeping in mind that they represent the phase-difference between two signals, rather than the phase of one or the other.

Given a set of *M* equally and linearly spaced phase bins with centers *H* = {φ_1_,…,φ_*M*_}, we define the 2D histogram of frequencies and phase-differences by:

(2.6) $${\hat{v}}_{a\,b}\,\left(f,\varphi \right)=\frac{1}{\left|T_{\varphi }\,\left(f\right)\right|}\,\sum_{t\in T_{\varphi }\,\left(f\right)}\left|v_{a\,b}\,\left(t,f\right)\right|$$

(2.7) $$T_{\varphi }\,\left(f\right)=\left\{t\in T|{\text{bin}}_{\varphi }\,\left(∠\,v_{a\,b}\,\left(t,f\right)\right)=1\right\}$$

where bin_φ_(*x*) returns *1* if *x* and φ are in the same bin, and 0 otherwise. *T*_φ_(*f*) is the set of all time samples such that ∠*v*_*ab*_(*t*,*f*) is in the bin containing φ.

If pairs of sensors are spaced sufficiently close together in the environment being recorded, then the cross-wavelet transform between sensors in such a pair allows us to measure propagating quasiperiodic activity. The sign of the phase-difference determines the direction of propagation. The value of the phase-difference φ (rad) at the frequency of interest *f* (Hz) and the separation between the pair of sensors *d* (cm) can be used to determine the apparent velocity of propagation *u* (cm/s) with the simple formula:

(2.8) u=d⁢2⁢π⁢f/φ

For quasiperiodic pressure signals in these data, a more appropriate measure of propagation may be “pace” which is the inverse velocity *u*^−1^ (s/cm), where synchronous events (or phase-locking) between the two signals may have a more robust-for-modeling pace of 0, rather than a velocity at ±∞.

## Statistical Framework

For each unit of statistical data, we obtain from the wavelet analysis a 1D curve $\hat{v}\,\left(f\right)$, or a 2D surface $\hat{v}\,\left(f,\varphi \right)$. Such a curve or surface is considered to be a response under the influence of a set of predictors which can be any number of categorical or numerical variables specified by the experimental design. We want to measure and compare the effects of the given predictors.

An independent regression model could be fit for each location *x* in either the frequency *x* ∈ *F* or frequency-phase *x* ∈ *F*×*H* domains. However, performing an independent fit at each location would require a multiple-comparison adjustment, and would fail to account for correlations between locations, effectively weakening the power of the analysis.

Instead, we capture correlations between locations by treating the response curves and surfaces as individual functions rather than simply collections of independent points. We model these functions as samples from Gaussian processes, which allow us to specify a formula for correlation between locations, without needing to specify a formula for the shape of the functions themselves.

A Gaussian process (GP) is a probability distribution with an infinite number of random variables, such that any finite set of variables form a multivariate Gaussian distribution. This is achieved by specifying a covariance kernel function *k*(*x*,*x*′), which when given a finite set of locations *x* ∈ {*x*_1_,…,*x*_*N*_} allows us to build an *N*×*N* covariance matrix Σ with elements Σ_*ij*_ = *k*(*x*_*i*_,*x*_*j*_). We have only finite data, and so the kernel function is evaluated only at the available data locations when fitting the GP. However, we can inspect the GP at any number of arbitrary locations in the kernel’s domain, hence the infinite nature of the model as a step beyond a multivariate Gaussian. An analogy is fitting a simple regression line. The line is fit only to a finite set of data, but once we have an intercept *b* and slope *a* we can define a function *y*(*x*) = *ax* + *b*, where y-locations can be calculated for any choice of x-locations, not just those for which we have data.

### Model

The latent GP function-on-scalar mixed-effect model we use can be written in the form:

(3.1) $$y_{i}\,\left(x\right)∼𝒢\,𝒫\,\left(\eta_{i}\,\left(x\right),\sigma_{i}\,\left(x,x^{\prime }\right)\right)$$

(3.2) $$\sigma_{i}\,\left(x,x^{\prime }\right)=\omega_{i}\,\left(x\right)\,\omega_{i}\,\left(x^{\prime }\right)\,\left(k_{\sigma }\,\left(x,x^{\prime }\right)+\sigma_{\epsilon }^{2}\right)$$

(3.3) $$\eta_{i}\,\left(x\right)={\text{X}}_{i}\,\beta \,\left(x\right)+{\text{Z}}_{i}\,\text{b}\,\left(x\right)+o_{\eta }$$

(3.4) $$\text{log}\,\left(\omega_{i}\,\left(x\right)\right)={\text{W}}_{i}\,\gamma \,\left(x\right)+{\text{U}}_{i}\,\text{u}\,\left(x\right)$$

where *𝒢𝒫* represents the Gaussian process distribution, *k*_σ_ is a kernel function describing the structured ω-standardized noise covariance, $\sigma_{\epsilon }^{2}$ represents unstructured ω-standardized noise variance, and *y_i* is the response function for observation *i* ∈ {1,…,*N*}. The responses are based on the transformed power $\text{log}\,\left(\hat{v}\right)$. The intuition behind the ω-standardized noise co/variance can be seen by rearranging the terms in equations (3.1) and (3.2) to:

(3.5) $$\frac{y_{i}\,\left(x\right)-\eta_{i}\,\left(x\right)}{\omega_{i}\,\left(x\right)}∼𝒢\,𝒫\,\left(0,k_{\sigma }\,\left(x,x^{\prime }\right)+\sigma_{\epsilon }^{2}\right)$$

which facilitates efficient inference by not requiring the structured residuals on the right-hand-side of equation (3.5) to be sampled, nor requiring a matrix inversion per observation. When evaluating the likelihood specified by equation (3.5), for the 1D case a simple Cholesky decomposition is sufficient, but for the 2D case an eigen decomposition is needed to separate the kernel functions from the unstructured noise $\sigma_{\epsilon }^{2}$ (see^1^ for a Stan model source code example).

In the mean specified by equation (3.3), **X** ∈ *ℝ*^*N*×*P*^ is a design matrix of *P* population-level predictors (a.k.a. fixed-effects) with **X**_*i*_ ∈ *ℝ*^1×*P*^ representing the row vector of predictors pertaining to observation *i*. β=(β_1_,…,β_*P*_) is a *P*×1 vector of iid latent GPs representing the *P* population-level effects. **Z** ∈ *ℝ*^*N*×*J*^ is a design matrix of *J* group-level predictors (a.k.a. random-effects). **b** = (*b*_1_,…,*b*_*J*_) is a *J*×1 vector of potentially correlated latent GPs representing the group-level effects. Depending on the experimental design, an optional offset term *o*_η_ is included in equation (3.3) which may be either set to the mean of all *y* as a way of centring the data, inferred to include a measure of variability in the centering, or given a different value per observation if some measure of exposure needs to be incorporated that would not otherwise fit as its own predictor in **X** or **Z**.

Analogous to the predictors **X** and **Z** for the mean, the matrices **W** ∈ *ℝ*^*N*×*Q*^ and **U** ∈ *ℝ*^*N*×*R*^ are respectively, the population-level and group-level predictors for the log standard deviation equation (3.4), with corresponding effects γ and **u**. An explicit offset term is missing here since such an offset is implicitly handled by the scale of *k*_σ_.

Each GP function in each vector of population-effects is given an iid prior:

(3.6) $$\beta_{p}∼𝒢\,𝒫\,\left(0,k_{\beta_{p}}\,\left(x,x^{\prime }\right)\right)$$

(3.7) $$\gamma_{q}∼𝒢\,𝒫\,\left(0,k_{\gamma_{q}}\,\left(x,x^{\prime }\right)\right)$$

However, for the vectors of group-effects functions we include correlations between functions via multivariate or multi-output GPs:

(3.8) $$b_{j}∼𝒢\,𝒫\,\left(0,{\left(\Sigma_{b}\right)}_{j,j^{\prime }}\,k_{b_{j}}\,\left(x,x^{\prime }\right)\right)$$

(3.9) $$u_{r}∼𝒢\,𝒫\,\left(0,{\left(\Sigma_{u}\right)}_{r,r^{\prime }}\,k_{u_{r}}\,\left(x,x^{\prime }\right)\right)$$

where Σ_**b**_ and Σ_**u**_ are covariance matrices dependent on the structure of the **Z** and **U** design matrices. These Σ matrices will generally be block-sparse, facilitating efficient computation.

The kernel functions *k*_{σ,β,γ,*b*,*u*}_(*x*,*x*′) and their parameters, also known as *hyperparameters* of the GPs, will be covered in the next subsection “Kernel Functions.”

The response functions *y_i*, and the design matrices **X**, **Z**, **W**, and **U** are the supplied “input” data. The vectors of functions β, **b**, γ, **u**, and hyperparameters, are to be estimated and correspond to “outputs” of the inference. The structure of the design matrices depends on the experimental design, and we find it easiest to derive the design matrices (also known as “model matrices”) based on formula notation as specified in section 2 of Bates et al. (2015). We provide an application in section “Results” using the formulae (3.15) and (3.16).

We are interested in modeling power that was calculated using the wavelet transform as described in sections “Wavelet Transform” and “Cross-Wavelet Transform.” To fit the power over frequencies, *x=f* is a scalar that represents frequencies. To fit over frequencies and phase-differences, *x* = (*f*,φ) is a 2D point that represents frequencies in one dimension and phase-differences in the other.

### Kernel Functions

The form and parameters of the kernel functions *k* depend on whether the response functions are 1D or 2D. There are many potential kernels to choose from, and they can even be built up from smaller kernels (Duvenaud, 2014), but for the sake of brevity we will limit our exposition to one concrete kernel function for each type of domain.

For the case of 1D curves over frequencies we use a log-space squared-exponential kernel:

(3.10) $$k\,\left(f,f^{\prime }\right)=\tau^{2}\,\text{exp}\,\left(-\frac{{\left|\text{log}\,\left(f\right)-\text{log}\,\left(f^{\prime }\right)\right|}^{2}}{2\,\lambda^{2}}\right)$$

with λ specifying the lengthscale of the correlation based on the distance |log(*f*)−log(*f*′)| between any two frequencies *f* and *f*′. At a distance of 0 we have equal frequencies *f* = *f*′, where the correlation is 1 and covariance is τ^2^. As the distance approaches ∞ the correlation and covariance approach 0.

For the case of 2D surfaces over frequencies and phase-differences we use a product of the log-space squared-exponential kernel and a periodic kernel:

$$k\,\left(f,\varphi ,f^{\prime },\varphi^{\prime }\right)=\tau^{2}\,\text{exp}\,\left(-\frac{{\left|\text{log}\,\left(f\right)-\text{log}\,\left(f^{\prime }\right)\right|}^{2}}{2\,\lambda_{f}^{2}}\right)$$

(3.11) $$\text{exp}\,\left(-\frac{2\,{\text{sin}}^{2}\,\left(\frac{1}{2}\,\left|\varphi -\varphi^{\prime }\right|\right)}{\lambda_{\varphi }^{2}}\right)$$

where λ_*f*_ and λ_φ_ specify the log-frequency and phase-difference lengthscales. When the difference in phase-differences φ and φ′ is either 0 or 2π, or any integer multiple of 2π, then the phase-difference component of the kernel will be 1, identifying the locations φ and φ′.

For equations (3.6) and (3.7), *k* represents the kernel function used in constructing a *covariance* matrix, but for equations (3.8) and (3.9) *k* is a kernel function used in constructing a *correlation* matrix by setting τ = 1, since including a free parameter for variance in *k* would make the model non-identifiable due to the variance parameters already defined in Σ_**b**_ and Σ_**u**_.

The kernel in equation (3.11) is separable, such that we can write it as:

(3.12) $$k\,\left(f,\varphi ,f^{\prime },\varphi^{\prime }\right)=k\,\left(f,f^{\prime }\right)\,k\,\left(\varphi ,\varphi^{\prime }\right)$$

(3.13) $$k\,\left(\varphi ,\varphi^{\prime }\right)=\text{exp}\,\left(-\frac{2\,{\text{sin}}^{2}\,\left(\frac{1}{2}\,\left|\varphi -\varphi^{\prime }\right|\right)}{\lambda_{\varphi }^{2}}\right)$$

where with abuse of notation we are identifying kernel functions based on their argument symbols, such that *k*(*f*,*f*′) and *k*(φ,φ′) are different functions, with *k*(*f*,*f*′) defined in equation (3.10) and *k*(φ,φ′) defined in equation (3.13). Since we can factorize the 2D kernel equation (3.12), we can create a covariance matrix using the Kronecker product of the individual covariance matrices built from kernels equations (3.10) and (3.13):

$$\begin{array}{ccc} \Sigma & = & \Sigma_{F}\,⊗\Sigma_{H}\\ {\left(\Sigma_{F}\right)}_{i\,j} & = & k\,\left(f_{i},f_{j}\right)\\ {\left(\Sigma_{H}\right)}_{i\,j} & = & k\,\left(\varphi_{i},\varphi_{j}\right) \end{array}$$

The Kronecker factorization of the kernel matrices also allows for a substantial speed up in the numerical calculation of the Cholesky and eigen decompositions of the covariance matrices (Saatçi, 2012) used in inference.

Prior distributions for hyperparameters λ and τ are experiment dependent, and will in general depend on the scale of the data. For the application presented in section “Results,” each σ,β,γ,*b*,*u* (subscript omitted for brevity) is treated independently, unless otherwise specified. We used λ∼Lognormal(0,1) with the exception λ_σ_∼Lognormal(−0.7,1) while ensuring λ_σ_ < λ_{*f*,φ}_. For the correlation between λ_*f*_ and λ_φ_ we used ρ_*f*φ_∼Beta(2,2), and τ∼Γ(2,1).

### Implementation

A coarse grid was chosen for the functional domain so that posterior sampling could complete within a reasonable time. The grid can be refined relatively quickly after the expensive sampling step. Rather than the naïve linear or cubic interpolation, we can use GP prediction such that the covariance between locations is faithfully preserved in the refinement.

Given a vector of *N* grid coordinates **x**, a vector of *M* refined coordinates **x**_∗_, a vector of *N* function values **y** corresponding to **x**, and the kernel function *k*, then we can produce a vector of *M* refined function values **y**_∗_ at **x**_∗_ with:

(3.14) $${\text{y}}_{*}=\Sigma \,\left({\text{x}}_{*},x\right)\,\Sigma \,{\left(x,x\right)}^{-1}\,\text{y}$$

where Σ(**x**,**x**) is the *N*×*N* covariance matrix obtained by applying *k* to the coordinates in **x**, and Σ(**x**_∗_,**x**) is the *M*×*N* matrix given by the covariances obtained by applying *k* to **x**_∗_ and **x**.

Note, for the 2D case we can take advantage of:

$$\begin{array}{ccc} {\left(\Sigma_{F}\,⊗\Sigma_{H}\right)}^{-1} & = & \Sigma_{F}^{-1}\,⊗\Sigma_{H}^{-1} \end{array}$$

We use the Hamiltonian Monte Carlo sampler from the Stan (Carpenter et al., 2017) package to obtain a posterior distribution of GPs which can be inspected to detect where and how locations may differ between various categorical predictors.

We apply the method to data (described in Section “Data”) recorded from the descending and sigmoid colon of 11 healthy volunteers and 12 patients with slow-transit constipation, during 1 h preprandial and postprandial periods. Using formula notation:

(3.15) η∼g⁢r⁢o⁢u⁢p*r⁢e⁢g⁢i⁢o⁢n*m⁢e⁢a⁢l+(r⁢e⁢g⁢i⁢o⁢n*m⁢e⁢a⁢l|s⁢u⁢b⁢j⁢e⁢c⁢t)

(3.16) log⁢(ω)∼group*region*meal+nchan

the design matrices **X** and **Z** are constructed from formula equation (3.15), and **W** and **U** from equation (3.16), according to the construction process described by Bates et al. (2015), where the log in equation (3.16) is a transformation of ω. The *group* predictor is a categorical variable indicating the group each subject belongs to: healthy or slow-transit constipation. The *region* predictor is a categorical variable indicating from which region of the colon the unit of data was recorded: descending or sigmoid. The *meal* predictor is a categorical variable indicating whether a recording was obtained during the preprandial or postprandial state, corresponding to a meal effect. The categorical variable *subject* identifies the subject.

The *nchan* predictor in equation (3.16) is a real-valued standardized count of the number of sensors (or channels) in the recording, which varies per subject and per region. When computing weighted-averages over time as specified in sections “Wavelet Transform” and “Cross-Wavelet Transform,” we average not only over time but over both time and channels by effectively flattening the wavelet results into a single channel of length *c*|*T*|, where *c* is the number of channels and |*T*| is the number of time samples. Fewer channels are expected to result in a greater variation in the global averages, which is why we included it as a confounding factor of the signal variance. We set **U** = 0 with the formula equation (3.16) since we don’t have repeated measurements, and so a within-subject variation is poorly identified.

Two types of responses were analyzed, given by the 1D and 2D power from equations (2.4) and (2.6). The power was log-transformed to obtain the *y*′s in model equations (3.1–3.4). For the 1D responses 33 frequency-bins were used, and for the 2D responses 17 frequency-bins and 18 phase-bins were used. After sampling from the posterior, the 1D responses were subdivided by a factor of 4 from 33 to 129 frequency^2^ bins, and the 2D responses were subdivided by a factor of 6 from 17 to 97 frequency-bins and 18 to 108 phase-bins via GP interpolation equation (3.14).

For each response type, the Hamiltonian Monte Carlo run consisted of 500 warm-up iterations and 500 sampling iterations over 8 (0-initialised) chains resulting in 4000 samples from the posterior distribution. We used an adapt-delta of 0.9. Diagnostics showed no divergent transitions, a top tree depth of 10, and visual inspection of trace plots showed good convergence that was validated by an $\hat{R}\approx 1$. Data appeared consistent with the posterior predictive distribution. On an i9-9900K processor running Windows 10 with 32GB RAM using PyStan v2.19.1.1 (Stan Development Team, 2019) with 8 parallel CPU cores (1 per chain), the 1D response type completed sampling in 75 min, and the 2D response type completed in 44 h. The computation process from raw pressure recording to time-averaged wavelet spectra for the 88 individual observations took approximately 30 s each.

## Data

We apply the aforementioned spectral decompositions and associated statistical analysis to colonic manometry data obtained to compare healthy volunteers and patients with slow-transit constipation. Pre-processing of the data was done to remove baseline drift and synchronous pressure increase removal in the same manner as detailed in Wiklendt et al. (2013). A synchronous pressure increase was defined as a synchronous increase in pressure waves that occurred across all manometry channels. Synchronous pressure waves that did not span all recording channels were not affected by this filtering. Pressures below 1mmHg were then clamped to 1mmHg, and log-transformed so that high-amplitude events would not overpower potentially interesting low-amplitude oscillations.

The details of the healthy subjects, constipated patients, catheter types, placement, protocols and data collection have been described in a previous publication (Dinning et al., 2015). These are summarized briefly below.

### Subjects

Colonic manometry was performed in 14 patients with scintigraphically confirmed slow transit constipation (2 male; median age 52 years; range 24–76 years). Colonic scintigraphy studies indicated that 13 of the 14 patients had >90% retention of isotope at 72 h. The remaining patient had no reading at 72 h but had >50% retention at 96 h. These data were compared to the colonic manometry recordings from 12 healthy adults (5 men; median age 51 years; range 27–69 years). Abdominal x-rays, taken at the end of each study, confirmed that the catheter tip was clipped to the ascending or hepatic flexure in 8 patients and to the transverse or splenic flexure in 6. In healthy subjects the catheter tip was located distal to or at the hepatic flexure in 11 and at the splenic flexure in 1. As all subjects had pressures sensors located in the descending and sigmoid colon, we used data from these regions for the analysis and results described in this article.

All participants in the study had given written, informed consent and the studies were approved by the Human Ethics Committees of the South Eastern Area Health Service, Sydney and the University of New South Wales (05/122; May 2010), and The Southern Adelaide Health Service / Flinders University Human Research Ethics Committee (419.10; March 2011).

### Colonic Manometry

Colonic manometry was recorded with a fiber optic catheter containing 72 sensors spaced at one-centimeter intervals. On the day prior to the manometric recording, the bowel was cleared using sodium picosulphate and polyethylene glycol (Pharmatel Fresenius Kabi Pty Ltd., Hornsby Australia). All subjects drank clear fluids overnight. Lying in the left lateral position, with conscious sedation using midazolam and fentanyl, the manometry catheter was introduced with a colonoscope and clipped to the mucosa using Endoclips (Resolution Clip^®^ Boston Scientific, MA, United States).

### Study Protocol

Recordings were commenced within 60 min of the subject waking after the catheter placement. After a 2-h basal recording period, all subjects were given a 700Cal meal (24% protein, 43% fat, 33% carbohydrate). The meal consisted of 300ml of TwoCal^®^ HN Vanilla (Abbott Nutrition, Columbus, OH, United States) and a chicken sandwich. Colonic pressures were then recorded for a further 2 h.

## Results

**FIGURE 2:**
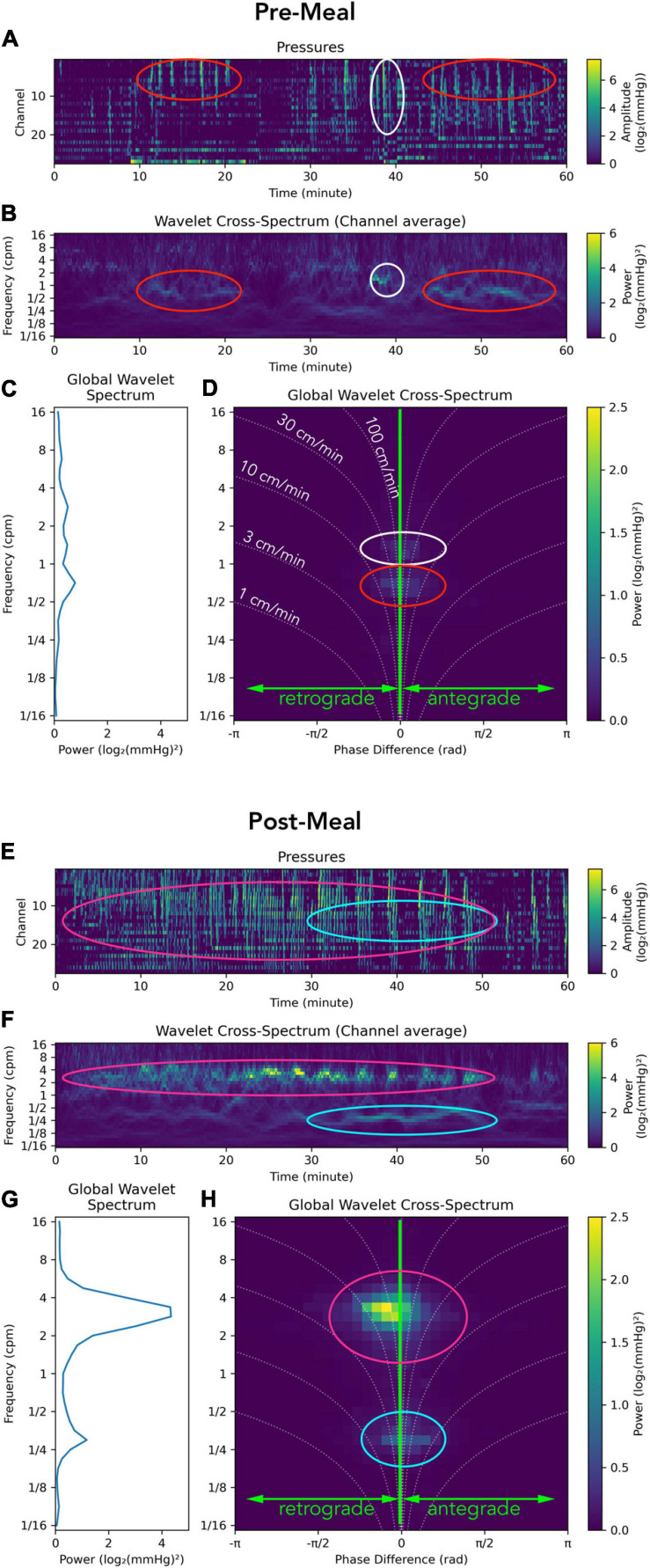
Representation of a manometry recording from the sigmoid colon in a single healthy adult prior to (top; **A–D**) and after a meal (bottom; **E–H**). Data from each study period is displayed using: (I) Color maps depicting raw pressure data from the 30 sensors within the sigmoid colon **(A,E)**. Higher amplitude events are colored pale green. (II) The power across the frequency range of 1/16th to 16 cycles per minute (cpm) is shown in panels **(B,F)**. (III) Graphs summarizing the power at each frequency are displayed in panels **(C,G)**. (IV) A summary of the 2D cross-wavelet analysis is depicted in panels **(D,H)**, where the vertical line at 0 on the x axis indicates synchronous activity. Retrograde propagation is displayed to the left of the midline, and antegrade to the right of the midline. The curved dotted lines indicate the speed of propagation, from 1 to 100 cm/min. The brightness of green pixels represents an increase in power. In this healthy adult prior to the meal, multiple frequencies were recorded, with no single frequency dominating **(C,D)**; while propagating activity at ∼1.5 cpm (white oval) and ½ cpm (red oval) exists, its power is so low that it is barely visible. The timing of this propagating activity is shown by white and red ovals in panel **(B)** and in the raw trace in panel **(A)**. After the meal there is a clear increase in the power of contractile activity [compare **(A,E)**], especially in the 2–4 cpm range **(G)**. This activity propagates in a predominantly retrograde direction **(H)**. The timing and location of this propagating activity can be seen in the red ovals in panels **(E,F)**. Another motor pattern emerges at ∼ 1/4 cpm after the meal [aqua ovals in panels **(E,F,H)**].

An example of the analysis applied to a recording from the sigmoid colon in a healthy adult is shown in Figure 2. The images contain the manometric traces constructed as PMaps (Figures 2A,E), the wavelet power spectrum of pressure waves at each moment (Figures 2B,F), the global wavelet power spectrum showing the dominant frequencies for the period (Figures 2C,G) and the global wavelet power cross-spectrum showing the dominant frequencies and their directions of propagation (Figures 2D,H).

In this example, the meal induced a large increase in power at 2–4 cpm (Figures 2F,G), which propagated mostly in a retrograde direction at 30–100 cm/min (Figure 2H; magenta oval). A second major frequency (∼1 every 3 min) also occurred 30–50 min after the meal (Figures 2E,F,H; aqua oval) and consists of individual clusters each containing pressure waves occurring at 2–4 cpm; clusters visible in Figure 2E.

### 1D Group Analysis

In this section we constructed power vs. frequency plots of motor events and compare them between healthy adults and patients in the descending and sigmoid colon.

#### Healthy Adults vs. Patients With Slow Transit Constipation; Descending Colon (Figure 3)

**FIGURE 3:**
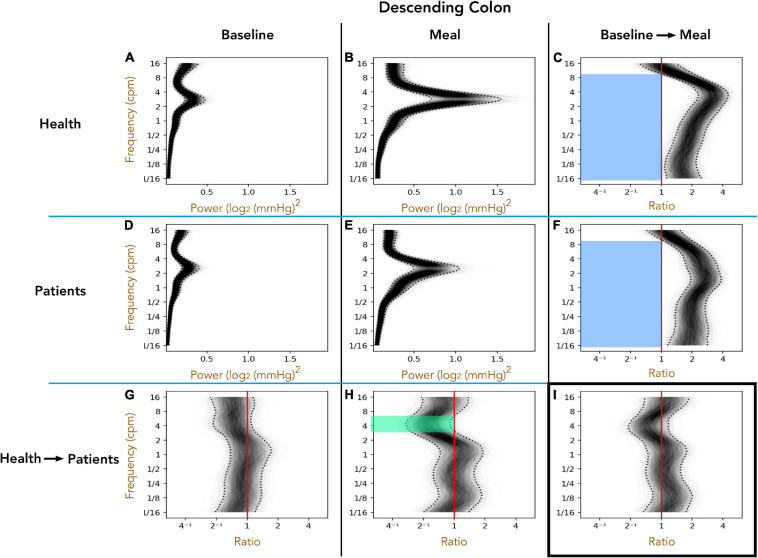
The one-dimensional (1D) analysis of pressure waves across a range of frequencies in the descending colon for healthy adults (top row; **A–C**) and patients with slow transit constipation (middle row; **D–F**) during the preprandial (left column; **A,D,G**) and postprandial (middle column; **B,E,H**). Comparisons between the two groups are shown on the bottom row **(G–I)**, and the ratio between periods on the right column **(C,F,I)**. In each image, frequency is shown on the *Y*-axis. In panels **(A,B,D,E)** power is shown on the *X*-axis. 2000 overlapping gray lines in each panel represent posterior samples, and the dotted black lines form envelopes of 95% credible intervals. Panels **(G,H)** represent the power ratio across the frequency range, between patients and healthy adults. When the entire envelope lies to one side of the vertical red line (which represents a ratio of 1), this shows a significant deviation. Thus, in the period after a meal, if we compare patients **(E)** with health in panel **(B)** you can see a significant reduction in power of the 3–6 cpm activity in the patients [shown by green area in panel **(H)** for the frequencies where the entire envelope lies to the left of the red vertical ratio line]. Panels **(C,F)** depict the ratio of power of postprandial activity to preprandial activity for healthy adults and patients, revealing that both groups show a significant increase in power in frequencies ranging from 1/16th cpm to 9 cpm (the envelope lies to the right side of the red vertical ratio line). Panel **(I)** shows that the pan-frequency increase in power did not differ significantly between patients and healthy adults.

The 1D analysis provides an indication of the power of pressure waves of different frequencies over a 1 h period. Preprandial activity is compared to the 1 h postprandial period. Furthermore, the difference between the periods can then be plotted to reveal significant changes caused by the meal, or significant differences between groups prior to or after the meal. During the preprandial recordings for healthy adults (Figure 3A) and patients (Figure 3D), a peak in power occurs at 2–4 cpm. A comparison in preprandial power between healthy subjects and patients is shown in Figure 3G. As can be seen there are no significant differences, indicated by the ratio between the two power densities not being outside of the 95% credible band (dotted curves). In the postprandial period, the peak at 2–4 cpm becomes more prominent (Figures 3B,E). The difference in post-meal activity between healthy subjects and patients is plotted in Figure 3H which shows that in patients, activity from 3 to 6 cpm is of lower power than in healthy subjects, as indicated by the ratios within the 95% credible band being all below 1 (green shaded region).

The effect of the meal (relative to preprandial activity) is shown in Figure 3C (healthy subjects) and Figure 3F (patients). In both groups the meal induced a significant increase in power across almost the full spectrum of frequencies tested (blue shaded regions in Figures 3C,F). In Figure 3I, comparison of the meal effect between the patients and healthy adults indicated no significant differences, as indicated by a ratio of 1 remaining within the 95% confidence band. This analysis clearly shows that patients displayed a reduced power in the frequencies between 3 and 7 cpm after the meal compared to healthy adults. However, a meal proportionally induces a similar increase in power across the range of frequencies in both groups.

#### Healthy Adults vs. Patients With Slow Transit Constipation; Sigmoid Colon (Figure 4)

**FIGURE 4:**
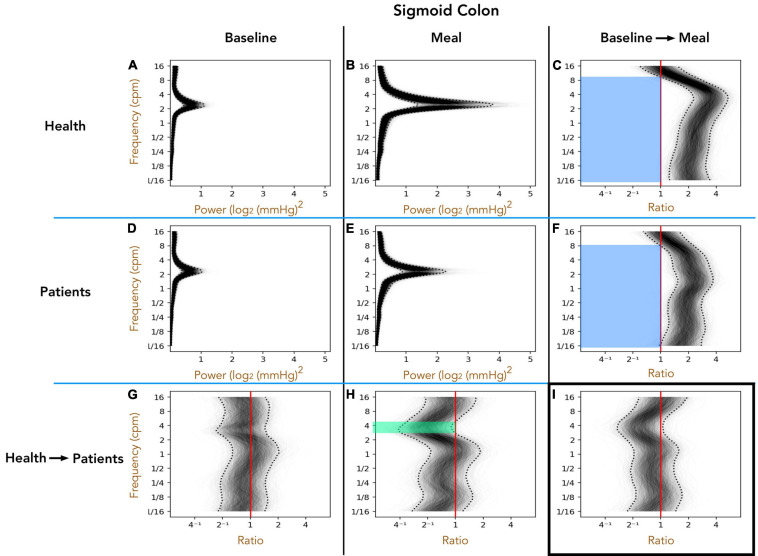
The one-dimensional (1D) analysis of pressure waves across a range of frequencies in the sigmoid colon of healthy adults (top row; **A–C**) and patients with slow transit constipation (middle row; **D–F**), during the preprandial (left column) and postprandial periods (middle column). The components of the figure are identical to those for Figure 3, apart from the region of bowel studied. In the postprandial period, the power of 3–6 cpm contractions were increased compared to preprandial, but this effect was smaller in the patient group compared to healthy adults [see green area in panel **(H)** for the frequencies where the entire envelope lies to the left of the red vertical ratio line]. The meal caused a significant increase in power at frequencies ranging from 1/16th cpm to 9 cpm [see blue areas **(C,F)**]. This overall effect of the meal did not differ between the groups **(I)**.

As with the descending colon, peak frequencies in the sigmoid colon were between 2 and 4 cpm, in both healthy adults and patients, in the pre- and postprandial periods (Figures 4A,B,D,E). The post-meal 3–5 cpm power is significantly reduced in patients when compared to healthy adults (green shaded region in Figure 4H). In both groups the meal induced a significant increase in power across almost the full spectrum of frequencies tested (blue shaded regions in Figures 4C,F).

#### Healthy Adults vs. Patients With Slow Transit Constipation; Synchronous Pressure Increase Included

To determine if the automated removal of the synchronous pressure increases had any impact upon these results we re-ran the analysis without any removal of data. The results remained unchanged (See Supplementary Figure 1), indicating that in these data removal of the synchronous pressure increases has no impact upon our findings in either descending of sigmoid colon.

### 2D Group Analysis

The data used in the 1D analysis can be re-analyzed using a 2D group analysis which illustrates the direction of propagation of pressure waves across the range of frequencies (1/16^th^ to 16cpm).

#### Healthy Adults vs. Patients With Slow Transit Constipation; Descending Colon (Figure 5)

**FIGURE 5:**
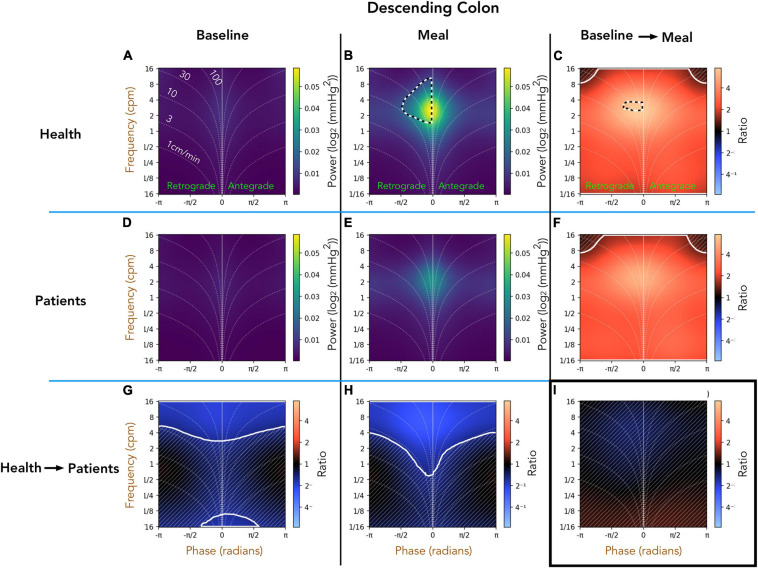
Two-dimensional (2D) analysis of propagating pressure waves in the descending colon at frequencies between 1/16th – 16 cpm. Panels **(A,B,D,E)** are identical in layout to Figures 2D,H. In panel **(B)**, the black and white hatched outline indicates that the power of retrograde 2–8 cpm propagating motor activity is significantly greater than the power of antegrade propagating contractions at the same frequency. The bottom row **(G,H)** compare power across the frequency range between patients and healthy adults during preprandial **(G)** and postprandial **(H)** periods. Blue regions in (bottom row; **G–I**) indicate activity of lower power in patients compared to healthy adults. The blue regions demarcated by the solid white line indicate frequencies that are significantly reduced in patients compared to healthy adults. The faint diagonal lines in panel **(G,H,C,F,I)** indicate regions of non-significance. Panels **(C,F)** compare power of propagating waves across the frequency range between preprandial and postprandial periods, for healthy adults and patients. The extensive red-shaded region in panels **(C,F)** indicates that propagating activity increased in power after the meal at all measured frequencies. The area marked by the solid white lines indicates a significant increase. Panel **(I)** compares the meal effect between patients and healthy adults, confirming that the comparative meal effect between the two groups was similar.

Comparison of the preprandial recordings between the two groups (Figures 5A,D) indicates a significant reduction in retrograde and antegrade propagation across a wide range of frequencies (4–16 cpm) in the patient group (ratios shown in Figure 5G). During the postprandial period in healthy adults, the retrograde cyclic activity between 2 and 8 cpm is of significantly greater power than antegrade cyclic activity at the same frequency (black and white hatched outline in Figure 5B). The propagated frequencies between 1 and 16 cpm were significantly reduced in patients compared to healthy adults during the meal period (pale blue area at top of Figure 5H). The effect of consuming a meal on propagation is shown in Figures 5C,F. In both groups, the meal caused a significant increase in all propagated frequencies, which did not differ between healthy adults and patients (Figure 5I). Therefore, while there were significant differences shown in the post-prandial motility between health and patients (Figure 5H), the proportional meal effect size was similar between the groups (Figure 5I).

#### Healthy Adults vs. Patients With Slow Transit Constipation; Sigmoid Colon (Figure 6)

**FIGURE 6:**
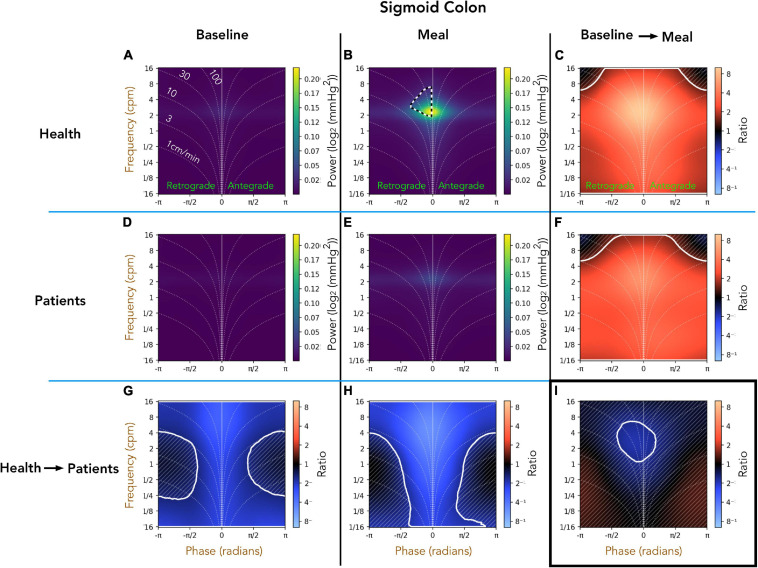
Two-dimensional (2D) analysis of propagating of pressure waves in the sigmoid colon at frequencies between 1/16th – 16 cpm. Panels **(A,B,D,E)** were constructed as in Figure 5. In panel **(B)**, the black and white hatched outline indicates that the power of retrograde propagating motor activity at 2–8 cpm was significantly greater than the power of antegrade propagating motor activity at the same frequency. Comparing the preprandial and postprandial periods for healthy adults and patients, the analysis shows that the power of propagating waves at all frequencies were reduced in patients **(G,H)**. The meal resulted in a significant increase in the power of waves at all frequencies in both groups (region demarcated by the solid white lines in panels **(C,F)**). Panel **(I)** shows that 2–6 cpm post-prandial increase in patients was significantly reduced compared to the increase in this frequency shown in healthy adults [region within the solid white outline in panel **(I)**].

A comparison of the preprandial recordings (Figures 6A,D) between the two groups indicates a significant reduction in both retrograde and antegrade propagation across the full range of frequencies in the patient group (Figure 6G; pale blue area). During the postprandial period in healthy adults, the retrograde cyclic activity between 2 and 8 cpm was of significantly greater power than the antegrade cyclic activity in the same frequency range (black and white hatched outline in Figure 6B). Comparison of the post-prandial period indicates a significant reduction in both retrograde and antegrade propagation across the full range of frequencies in the patient group (Figure 6H; pale blue area). The meal effect on propagation within each group is summarized in Figures 6C,F. In both groups, the meal caused a significant increase in the power of propagating activity at all frequencies, with a peak effect at 2–6cpm in healthy adults (Figure 6C; bright orange region). The meal also caused a significant increase in the power of propagating activity with a frequency between 2 and 6 cpm in patients (Figure 6F), but this increase was not as marked as in healthy adults (Figure 6I; blue region within the white circle).

#### Comparison Against Manual Analysis

Our original publication of these data used manual analysis to identify propagating motor patterns in healthy adults (Dinning et al., 2014). That article was the first to describe in detail the propagating motor pattern which consisted of pressure waves with a frequency of 2–6/min. This motor pattern was labeled the *cyclic motor pattern* and the key findings in that article, centered upon this motor pattern, included; (i) the cyclic motor pattern made up 69% of all propagating activity. (ii) It propagated in predominately retrograde direction. (iii) A meal was shown to increase the count of all motor patterns however, the major effect of a meal upon colonic motility was a significant (*P* < 0.001) increase in retrograde cyclic motor pattern. With our novel, automated technique, we have also shown at after a meal the retrograde cyclic activity between 2 and 8 cpm is of significantly greater power than antegrade cyclic activity at the same frequency [see sections “Healthy Adults vs. Patients With Slow Transit Constipation; Descending Colon (Figure 5)” and “Healthy Adults vs. Patients With Slow Transit Constipation; Sigmoid Colon (Figure 6)”]. The meal also resulted in a significant increase power of all propagating activity, with a peak effect at 2–6 cpm in healthy adults.

In our follow-up article, comparing the data from healthy controls to patients with slow transit constipation, our manual analysis showed that a meal induced a significant increase in the cyclic motor pattern in patients, but the increase was significantly reduced in comparison to increase observed in healthy adults (Dinning et al., 2015). These findings are confirmed in this current article (See Figure 6I).

## Discussion

In this article, we have presented a method for analyzing high-resolution, spatiotemporal colonic manometry data by computing various time-averaged spectra and using them as responses in a functional mixed-effects model, inferred via Hamiltonian Monte Carlo. This approach has allowed us to identify the frequencies of colonic pressure waves and compare differences in their characteristics between healthy adults and patients with slow transit constipation. Our main findings indicate that; (i) in both groups, prior to and after a meal, the dominant frequency of pressure waves in the descending and sigmoid colon is between 2 and 6 cpm and a meal results in a significant increase in the power of pressure waves across a wide range of frequencies (1/16 – 8 cpm); (ii) in healthy adults only, the retrograde cyclic activity between 2 and 8 cpm is of significantly greater power than antegrade cyclic activity at the same frequency; (iii) in the sigmoid colon, the meal induced an increase in the power of antegrade, synchronous, and retrograde propagating activity with frequencies between 2 and 6 cpm, which was of significantly greater power in healthy adults than in patients.

Previously we had presented our first step in the computerized development of software for the analysis of colonic pressure waves (Wiklendt et al., 2013). That work allowed us to separate patients with slow transit constipation from healthy adults on the basis of a single “indicator value” calculated from the colonic manometry data. However, that indicator value provided no information on the frequency of pressure waves, or their direction and speed of propagation; all features provided by our current automated approach. In addition, we have also previously used fast Fourier transform (FFT) and wavelets to demonstrate a postprandial increase power of colonic activity (Dinning et al., 2015, 2016), but those publications lacked the rigorous statistical analysis of the current article. The advantages of the wavelet transform over the Fourier transform for our application are two-fold. Firstly, the wavelet transform provides an instantaneous spectrum at each time point, allowing us to remove harmonic artifacts with the MesaClip algorithm (Wiklendt et al., 2020), and also facilitating the comparison of spectra between adjacent sensors for each time point to obtain the cross-wavelet transform which can reveal propagation delays via phase differences. Secondly, although the short-time Fourier transform (STFT) could be used to compute near-instantaneous spectra, it requires one to choose (a) a window width, (b) a window function, and (c) an overlap amount between adjacent-in-time windows. The wavelet transform only needs the equivalent of (b), whereas the window width adjusts naturally to each frequency being analyzed, with the equivalent of maximum possible overlap without the prohibitively high computational burden as would be the case for the STFT.

Importantly, the outcomes of the colonic manometry analysis provided in this article do not contradict our manual analysis of these same data published previously (Dinning et al., 2014, 2015). To obtain the results in this article, less than two days were required. In comparison the manual analysis of the control and patient data took five weeks to perform. Thus, the detailed analysis provided by our automated technique is orders of magnitude beyond the methods currently available in both detail, speed of analysis, and manual labor saved.

The cyclic nature of colonic pressure waves shown in this analysis is not a new finding. Indeed, regular human rectal pressure waves at approximately 2–3/min were reported in Welch and Plant (1926). Nearly all colonic manometry studies since then show figures or report findings of pressure waves with similar frequencies. The physiological role of such motor patterns remains undetermined, but it is likely to play a role in mixing or retarding colonic flow (Spriggs et al., 1951; Rao and Welcher, 1996; Rao et al., 2001a; Lin et al., 2017a,b; Pervez et al., 2020). The frequency of 2–6 cpm is approximately the same as the frequency of human colonic slow waves (Rae et al., 1998; Carbone et al., 2013) which are generated by the interstitial cells of Cajal (ICC) (Huizinga et al., 2011; Costa et al., 2013). The rapid increase (within 60 s) in this motor pattern after a meal is commenced suggests that the slow wave activity can be modulated by extrinsic neural pathways. Therefore, the ability to accurately identify this motor pattern and determine the influence of physiological stimuli upon it may help to unravel both the normal physiology of healthy adult colonic motility and provide insight into abnormalities that exist in patients with functional colonic disorders.

**FIGURE 7:**
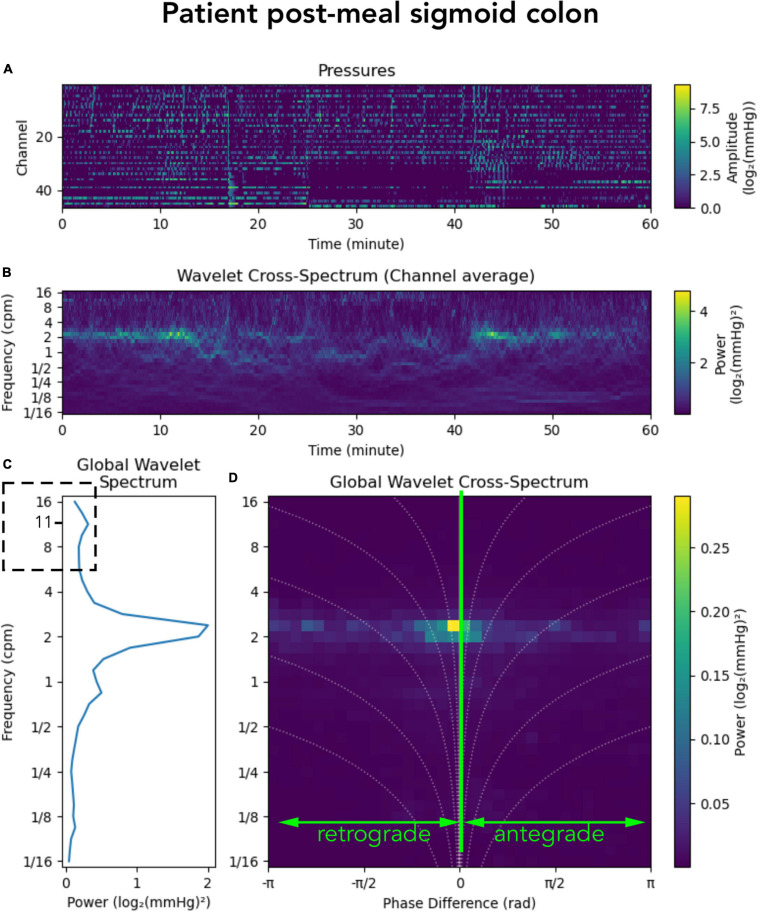
Representation of a manometry recording from the sigmoid colon in a single patient after a meal. **(A)** Shows the color maps depicting raw pressure data from the sensors within the sigmoid colon. **(B)** shows the power across the frequency range of 1/16th to 16 cycles per minute (cpm). **(C)** Shows graphs summarizing the power at each frequency; and **(D)** shows a summary of the 2D cross-wavelet analysis with retrograde and antregrade propagation. Synchronous activity is shown at 0 on the *x*-axis. Note that in panel **(C)** a peak can be seen at ∼11 cpm (hatched box). This higher frequency was seen in some of the subjects in this study, but the power of this frequency is very low.

In addition to the 2–6 cpm activity, a recent publication by Pervez et al. (2020) showed a cyclic motor pattern consisting of clusters of pressure waves at a frequency of 11–13 cycles/min. This motor pattern was identified throughout the colon and it occurred in isolation of other motor patterns or following high-amplitude propagating contractions. In our grouped data, a motor pattern of this frequency was not prominent either before or after a meal. However, that does not mean this higher frequency did not exist. Examples can be found in some of the individual subjects. Figure 7 shows post-meal sigmoid colon data from an individual patient in which a peak in the global wavelet spectrum can be seen at ∼11/min (Figure 7C; hatched box). The overall diminished prominence of this frequency in our data, compared to the study by Pervez et al. (2020) may reflect the different protocols used to record colonic motor patterns. In our data, manometry was recorded in a prepared colon (faces removed) with a fiber-optic catheter and, apart from a meal, no other stimulation was provided. The protocol used by Pervez et al., also recorded from a prepared colon, however, they used water-perfused manometry, colonic balloon distension, gave the subjects a meal and the laxatives prucalopride and bisacodyl. This combination of colonic stimulation may have initiated prominent 11–13 cycles/min motor activity. This highlights that differences in protocols should always be considered when comparing data between colonic manometry studies.

A common feature of many colonic manometry recordings is the high amplitude propagating contraction (HAPC). These events are associated with movement of content (Cook et al., 2000), defecation (Herbst et al., 1997; Bampton et al., 2000) and have been shown to be diminished or absent in patients with constipation (Bassotti et al., 1988; Rao et al., 2001b; Dinning et al., 2010). Therefore, their presence or absence in a manometry recording is always noted. The approach described in this article does not specifically identify these motor patterns, however, if several occur sequentially within the 1/16 – 16cpm range used in this analysis they will form part of the calculated result. This should not be seen as a problem, in short duration recording within the prepared colon, HAPC make up <2% of the propagating activity, with many healthy adults not having any (Dinning et al., 2014). For specific characteristics of the HAPC we would still recommend manual analysis.

In addition to HAPCs, articles on colonic manometry provide counts of all other propagating contractions and their extent of propagation. Such data is not available with this automated approach. However, software to both count the number of individual propagating contractions and calculate their propagation length, has been developed and validated by our colleges in New Zealand (Paskaranandavadivel et al., 2018). This work is currently submitted for publication elsewhere. It is likely that a combination of both approaches will be used in the future to provide a full description of colonic motor patterns.

It is also important to note that we have based our findings upon these data after we removed synchronous pressure increases that occurred across all recording channels. Recently there have been publications in which these synchronous pressure increases have been included in the analysis (Corsetti et al., 2017; Chen et al., 2018; Pervez et al., 2020). However, synchronous pressure increases can also be caused by abdominal strain, diaphragmatic movement (laughing), coughs, sneezes or by body movement. (Corsetti et al., 2017) discriminated between synchronous pressure waves caused by colonic motor activity and abdominal wall muscle activity using abdominal wall electromyography (EMG). In our study, EMG was not used and therefore we had no way of discriminating between artifact and a genuine colonic motor pattern. As such our pre-processing of the data prior to analysis involved the identification and removal of the activity. However, as shown in Supplementary Figure 1, such removal had no impact upon our findings. Synchronous pressure waves that did not span the entire recording length were always part of our data and can be seen in the 2-D images as the activity recorded at phase 0 (Figures 2D,H, 5, 6, 7D). Whether or not our automated approach improves the diagnostic potential of colonic manometry, remains to be determined, as yet it has only been performed on a small number of studies. As shown in Figure 2, our new approach does allow for a rapid appreciation of the colonic contractile activity in any given recording. Within 30 seconds figures can be produced which show the dominant frequencies of pressure waves, their propagation direction and speed and whether or not a meal (or any other stimulus) changes these characteristics. We are currently in the process of applying this analysis to larger data sets, with different colonic stimulation techniques and differing types of constipation. Such analysis may then allow us to determine whether defined categories of constipation (slow transit, normal transit, constipation predominant irritable bowel syndrome) display characteristic differences compared to healthy adults. Importantly this analytical approach will also allow us to determine the effects of treatment upon colonic motility.

## Data Availability Statement

The raw data supporting the conclusions of this article will be made available by the authors, without undue reservation.

## Ethics Statement

The studies involving human participants were reviewed and approved by the Human Ethics Committees of the South Eastern Area Health Service, Sydney and the University of New South Wales (05/122; May 2010), and The Southern Adelaide Health Service/Flinders University Human Research Ethics Committee (419.10; March 2011). The patients/participants provided their written informed consent to participate in this study.

## Author Contributions

LW devised the methodology and wrote the software and technical parts of the manuscript. PD wrote the manuscript. MC and PD established the concepts. MC, SB, and SS made critical revision of the manuscript for important intellectual content. All authors contributed to the article and approved the submitted version.

## Conflict of Interest

The authors declare that the research was conducted in the absence of any commercial or financial relationships that could be construed as a potential conflict of interest.

## Footnotes

1 https://github.com/lwiklendt/gp_kron_stan

2 Subdivision over frequencies is performed in log-space with *n*′=(*n*−1)*s* + 1 where *n*′ is the new bin count, *n* is the old bin count, and *s* is the subdivision factor.

## Supplementary Material

Supplementary Figure 1 The one-dimensional (1D) analysis of pressure waves across a range of frequencies in the descending and sigmoid colon for healthy adults and patients with slow transit constipation during the preprandial and postprandial periods. The left hand images (pan-recording synchronous pressure waves removed) are same as shown in Figures 3, 4 in the manuscript. The right hand images show the results of the analysis without the pan-recording synchronous pressure waves removed. Removal of the synchronous pressure waves that span all recording sites has no impact upon the final results.
