# Supplementary figures and images for: Automated Analysis Using a Bayesian Functional Mixed-Effects Model With Gaussian Process Responses for Wavelet Spectra of Spatiotemporal Colonic Manometry Signals
Source: Front Physiol. 2021 Feb 11;11:605066. doi: 10.3389/fphys.2020.605066 (PMC7905106; doi:10.3389/fphys.2020.605066)

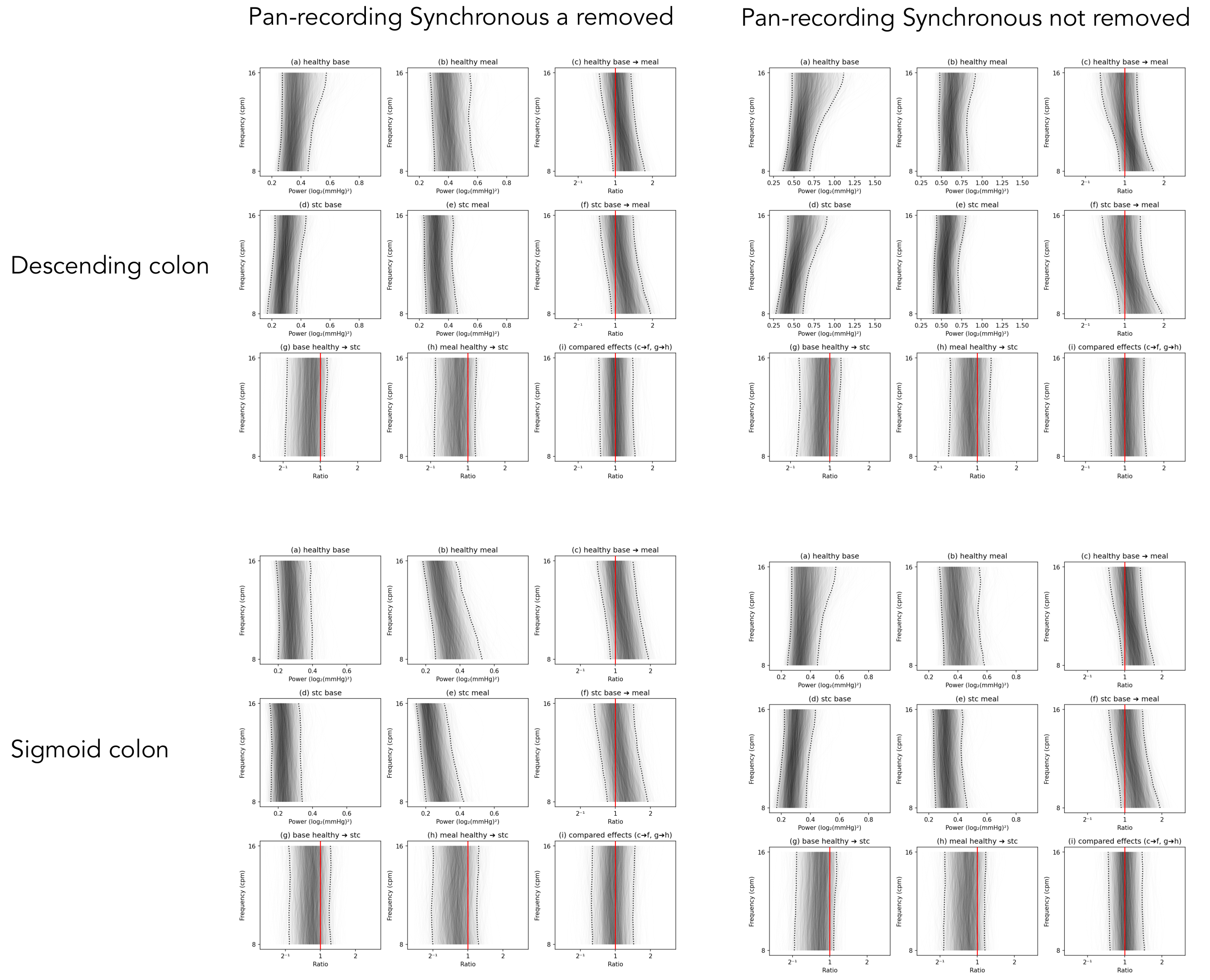

Supplement: Supplementary Figure 1 — The one-dimensional (1D) analysis of pressure waves across a range of frequencies in the descending and sigmoid colon for healthy adults and patients with slow transit constipation during the preprandial and postprandial periods. The left hand images (pan-recording synchronous pressure waves removed) are same as shown in Figures 3, 4 in the manuscript. The right hand images show the results of the analysis without the pan-recording synchronous pressure waves removed. Removal of the synchronous pressure waves that span all recording sites has no impact upon the final results. [file Image_1.TIF]
